# Dysfunction of Bone Marrow Vascular Niche in Acute Graft-Versus-Host Disease after MHC-Haploidentical Bone Marrow Transplantation

**DOI:** 10.1371/journal.pone.0104607

**Published:** 2014-08-13

**Authors:** Yonghua Yao, Xianmin Song, Hui Cheng, Gusheng Tang, Xiaoxia Hu, Hong Zhou, Jianmin Wang

**Affiliations:** Department of Hematology, Institute of Hematology, Changhai Hospital, Shanghai, China; University of Minnesota, United States of America

## Abstract

Acute graft-versus-host disease (aGvHD) is the most common complication of allogeneic hematopoietic stem cell transplantation (HSCT), which is often accompanied by impaired hematopoietic reconstitution. Sinusoidal endothelial cells (SECs) constitute bone marrow (BM) vascular niche that plays an important role in supporting self-renewal capacity and maintaining the stability of HSC pool. Here we provide evidences that vascular niche is a target of aGvHD in a major histocompatibility complex (MHC)–haploidentical matched murine HSCT model. The results demonstrated that hematopoietic cells derived from GvHD mice had the capacity to reconstitute hematopoiesis in healthy recipient mice. However, hematopoietic cells from healthy donor mice failed to reconstitute hematopoiesis in GvHD recipient mice, indicating that the BM niche was impaired by aGvHD in this model. We further demonstrated that SECs were markedly reduced in the BM of aGvHD mice. High level of Fas and caspase-3 expression and high rate of apoptosis were identified in SECs, indicating that SECs were destroyed by aGvHD in this murine HSCT model. Furthermore, high Fas ligand expression on engrafted donor CD4^+^, but not CD8^+^ T cells, and high level MHC-II but not MHC-I expression on SECs, suggested that SECs apoptosis was mediated by CD4^+^ donor T cells through the Fas/FasL pathway.

## Introduction

Allogeneic hematopoietic stem cell transplantation (HSCT) has been considered as one of the effective treatment strategies for hematological malignancies and other benign hematological disorders. [1] For patients who undergo allogeneic HSCT, acute graft-versus-host disease (aGvHD) is the most common complication that may lead to target organ damage. In those patients, impaired hematopoiesis has been associated with a poor prognosis. [2]–[4]

The mechanisms for hematopoietic dysfunction after aGvHD are still not fully understood. It was previously suggested that hematopoietic suppression might be mediated by inhibitory cytokines, such as TNF-α, produced during aGvHD as part of “cytokine storm”, or by a deficient bone marrow (BM) microenvironment (niche) damaged by conditioning reagents and/or by cytokines. Two types of BM niches were identified in recent years. Endosteal niche is mainly located in the endostium and composed of osteoblast cells; while vascular niche is formed with sinusoidal vascular endothelial cells (SECs) and perivascular cells.[5]–[8] Both endosteal and vascular niches play important roles in regulating self-renewal capacity and maintaining the stability of hematopoietic stem cell (HSC) pool.[9]–[19] Recently, Shono et al reported that, in an MHC-mismatched murine HSCT model, GvHD does not directly affect HSCs but rather targets osteoblast cells, leading to BM endosteal niche failure to support hematopoiesis reconstitution after HSCT.[20] It is known that the major targets of aGvHD, liver, skin, and intestinal tract, are characterized by being covered with endothelial cells. We hypothesized that vascular niche, mainly composed of SECs, might be the target of aGvHD. Further, the dysfunction of vascular niche may play an important role in hematopoietic impairment in aGvHD. In this study, we investigated the effect of aGvHD on viability and functions of vascular niche, and its impact on hematopoietic reconstitution in an MHC haploidentical matched aGvHD mouse model.

## Materials and Methods

### Mice

Male C57BL/6 (B6; H-2^b^, CD45.2^+^) mice and female BALB/C (H-2^d^, CD45.2^+^) were purchased from Silaike Company (Shanghai, China). Male CB6F1 (F1; C57BL/6×BALB/C, H-2^b/d^, CD45.1/2^+^) mice were purchased from Vital River Laboratories (Beijing, China). Male B6.SJL (H-2^b^, CD45.1^+^) mice were donated kindly by Professor Tao Cheng (Institute of Hematology, Chinese academy of medical science). Mice used for experiments were 6 to 8 weeks old at the time of HSCT. All mice were housed in a specific pathogen-free condition at Animal Facilities of Second Military Medical University (Shanghai, China). All mice had access to autoclaved water with 0.3% enrofloxacin. All animal experimental protocols were reviewed and approved by the Second Military Medical University ethics committee.

### Hematopoietic stem cell transplantation

To evaluate the effects of aGvHD on hematopoiesis, a MHC-haploidentical matched murine model of aGvHD was established. BM cells were prepared from femurs and tibiae of B6.SJL (CD45.1) donor mice. They were washed and resuspended in Dulbecco's modified Eagle's medium (DMEM, Invitrogen, CA, US) before injection. Donor splenocytes (SCs) were prepared with red blood cells removed by hypotonic lysis using red blood cell buffer (BD Pharmingen, CA, USA). Recipient mice (CB6F1, CD45.1/2) were exposed to a single dose of 800cGy total body irradiation (TBI). 4 hours within TBI, recipient mice were injected intravenously with either PBS (PBS group), BM cells (5×10^6^/mouse, BMT group), or BM cells (5×10^6^/mouse) plus SCs (6×10^7^/mouse) from the donors (GvHD group, n = 20/each group). 14 days after transplantation, CD45.1/2 and CD45.1 expression on hematopoietic cells was measured to confirm successful engraftment. The clinical manifestations of aGvHD were monitored after transplantation, which included weight loss, posture (hunching), activity, fur texture, and skin integrity. [21]–[23]The death of mice was recorded each day for survival analysis.

To evaluate the effects of aGvHD on hematopoietic cells, continuous transplantation and competitive transplantation were conducted to evaluate self-renewal and differentiation capacity of HSCs in the GvHD and BMT mice. Specifically, in the continuous transplantation model, 14 days after first transplantation, CB6F1 mice from the GvHD and BMT groups were sacrificed. BM cells (CD45.1) were obtained and transplanted into healthy C57BL/6 (CD45.2) recipients (5×10^6^/mouse), GvHD→ C57BL/6 and BMT→ C57BL/6 group, respectively. All recipient mice received a single dose of 800cGy TBI. In the competitive transplantation model, 14 days after the first transplantation, CB6F1 mice from the GvHD and BMT groups were sacrificed. BM cells (CD45.1) were obtained (2.5×10^6^/mouse) and mixed with equal amount of BMs that were obtained from healthy C57BL/6 (CD45.2) mice (2.5×10^6^/mouse). A total number of 5×10^6^ BM cells were transplanted into healthy C57BL/6 (CD45.2) recipients, GvHD/BM→ C57BL/6 and BMT/BM→ C57BL/6 group, respectively. All recipient mice received a single dose of 800cGy TBI.

To evaluate the effect of aGvHD on BM niche in the recipient mice, 14 days after first transplantation, recipient mice in the GvHD and BMT groups received a continuous transplantation from healthy C57BL/6 (H-2^b^, CD45.2^+^)BM (5×10^5^/mouse), C57BL/6→GvHD and C57BL/6→BMT group, respectively. One day before transplantation, all recipient mice had 200cGy TBI.

### Hematopoietic profiling, Flow cytometric analysis, cell sorting and cell counting

Blood samples from recipient mice were collected every 3 days since day 4 after transplantation. White blood cell (WBC), hemoglobin (Hgb), and platelet counts were monitored to evaluate hematopoietic reconstitution. Blood samples from recipient mice were collected every 3 days since day 4 after transplantation. White blood cell (WBC), hemoglobin (Hgb), and platelet counts were monitored to evaluate hematopoietic reconstitution. Recipient mice were humanly euthanized 14 and 21 days after transplantation. Donor derived hematopoiesis was analyzed. Basically, BM cells were harvested by repeated flush of single tibia by PBS for analysis. Commercial monoclonal antibodies CD3, CD4, CD8, CD11b, CD48, CD45.1, CD45.2, CD150, Sca-1, vascular endothelial growth factor receptor 2 (VEGFR2), VEGVR3, CXCR4, B220, Gr-1, Ter119, Ki-67, PI, Annexin V, Fas, MHC-I, MHC-II, FasL (BD company, Ebiosciences, CA, USA) and caspase-3 (Sanza Cruz Biotechenology, USA) were applied for analysis. Two-, three-, or four-color flow cytometry (BD company, USA) was performed to measure the surface expression of molecules according to standard techniques. Background staining for antibodies was performed in negative cell lines and with matched fluorochrome-conjugated isotype controls. The stained cells were incubated with mAbs for 20 min at 4°C, washed with PBS twice, resuspended in PBS, and analyzed on a flow cytometer with Cell Quest software.

Mouse lineage mixture: CD3, CD11b, Gr-1, B220, and Ter-119 antibodies were used to sort HSCs. VEGFR2^+^/VEGFR3^+^/Sca-1^−^ were used to sort SECs. The purity of sorted cells was routinely more than 95%.

The number of BM MNCs (Mononuclear cells) per tibia was calculated with hemocytometer. The percentages of B lymphocytes (B220), granulocytes (Gr-1), monocytes (CD11b), CD45.1/CD45.2 cells, and Lin^−^CD48^−^CD150^+^ (HSCs) cells were monitored by flow cytometry. The percentage of SECs was detected by flow cytometry with VEGFR2^+^/VEGFR3^+^/Sca-1^−^ phenotype. CXCR4^+^ expression on Lin^−^CD48^−^CD150^+^ cells were also evaluated 14 days after transplantation. The absolute counts of every kind of cells were calculated with total MNC number per tibia and their percentages.

### Quantitative analysis of transcription factor gene expression by real-time polymerase chain reaction

BMs from the recipient mice were harvested at day 14 and day 21. 5−8×10^5^ SECs were sorted with VEGFR2^+^/VEGFR3/^+^Sca-1^−^ gating, 4−6×10^5^ HSCs were sorted with Lin^−^/CD48^−^/CD150^+^ gating, 10^6^ CD4^+^/CD8^−^ and CD4^−^/CD8^+^ T cells were sorted with CD8/CD4 gating as described previously. Total RNA was extracted using Trizol (Invitrogen, CA, USA) according to the manufacturer instructions. The extracted RNA samples were used for cDNA transcription, which then were used as template for real-time quantitative polymerase chain reaction (PCR) (Biosystems 7300 Fast Real-Time PCR system, USA). The primers used for PCR reaction are shown in table S1. Relative levels of expression were determined using a housekeeping gene GAPDH. The relative expression of Fas or other genes is relative to its normal control (NC) group, according to 2^−ΔΔCT^ formula:  = 2-^[ΔCT(Fas)- ΔCT(NC)]^ = 2-^{[(Fas)CT-(GAPDH)CT]-[(NC)CT-(GAPDH)CT]}^.

### ELISA assay

14 and 21 days after transplantation, vascular endothelial growth factor (VEGF) levels were evaluated by enzyme-linked immunosorbent assay (ELISA) kit (eBioscience, San Diego, CA, US) according to manufacturer's instructions. In addition, single tibia of GvHD and BMT group mice was repeatedly flushed by constant volume of 1ml PBS. Supernatant was collected. VEGF were analyzed in the serum and supernatant samples. Samples and controls were run in duplicate. The absorbance at 450 nm was measured using a Thermomax microplate reader (Bio-Rad, Rockaway, NJ, USA).

### Histological analysis and immunohistochemistry staining

14 and 21 days after transplantation, aGvHD target organs, including liver, skin and intestines, were obtained and formalin preserved, paraffin embedded, sectioned, and hematoxylin and eosin (H&E) stained. Pathologic assessment of degree of inflammation was made by a pathologist unaware of the origin of the sections.

Femurs were decalcified using Decalcifying Solution (Richard-Allan Scientific, MI) and embedded in paraffin. Paraffin sections were stained with H&E. For detection of VEGFR3, paraffin sections were antigen retrieved. After endogenous peroxidase and non specific protein block (5% BSA, 10% goat serum, 0.02% Tween-20), anti-VEGFR3 mAb (BD) was incubated overnight at 4°C. After secondary polyclonal antibody and streptavidin horseradish peroxidase incubations (Jackson IR, PA), staining was developed with DAB and briefly counterstained in Mayer's hematoxylin (DAKO).

### Statistical analysis

Survivals in different groups were evaluated using Kaplan-Meier estimated event rates (log rank test) with SPSS 11.5 software. Group comparisons were performed using the unpaired two-tail Student t-test. Data were shown as mean ± standard deviation for separate experiments. A *P* value of <0.05 was considered statistically significant.

## Results

### Hematopoietic suppression and recovery in MHC-haploidentical matched murine model of aGvHD

**Figure 1:**
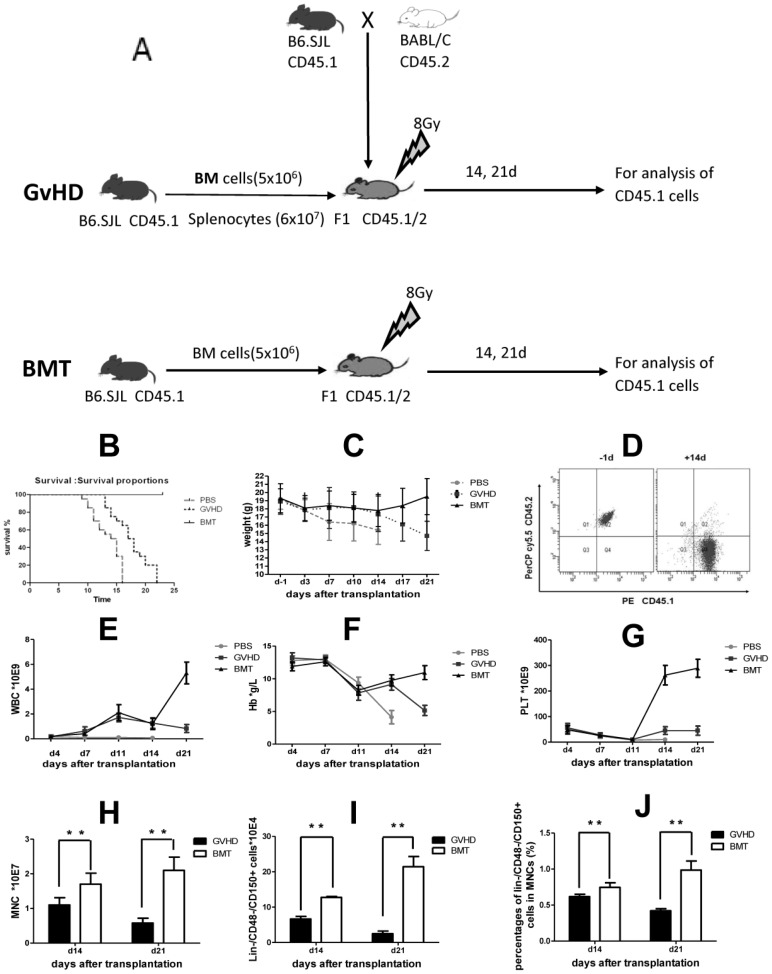
MHC-haploidentical murine GvHD model and suppression of hematopoiesis during GvHD. (A) A MHC-haploidentical murine GVHD model was established by transplanting BM cells (5×10^6^) plus splenocyts (6×10^7^) from B6.SJL donor mice (CD45.1) into lethally irradiated CB6F1 recipient mice (CD45.1/2) [B6.SJL (CD45.1)→CB6F1 (CD45.1/2)]. Recipent mice received BM cells alone as control groups. (B) Survival of mice receiving HSCT with donor BM plus splenocytes or donor BM only (*P*<0.05, Log-rank test). (C) Body weight of mice receiving HSCT (N = 20 in each group on day 3; n = 4 in GvHD and n = 20 in BMT respectively on day 21 post-transplantation). (D) Engraftment of donor-derived cells after HSCT in a GvHD mouse. (E–G) Kinetics of WBC, Hgb, and platelet counts after HSCT. (H) MNCs count on day 14 and day 21 after HSCT. (I) Count of Lin^−^/CD48^−^/CD150^+^ cells after HSCT. (J) Percentage of Lin^−^/CD48^−^/CD150^+^ cells in MNCs after HSCT. Data are shown as mean ± SD and from 1 of 3 experiments with similar results. *P<0.05; **P<0.01 (n = 4, *t*-test).

An MHC-haploidentical matched murine model of aGvHD was established to evaluate the effects of aGvHD on hematopoiesis in CB6F1 (CD45.1/2) mice transfused with BM cells plus splenocytes from B6.SJL CD45.1 donor mice after TBI (**Figure 1A**). Control BM transplant (BMT) mice received BM alone after TBI, and blank mice (PBS) received no BM transplantation after TBI. All mice in the PBS group died within 16 days after transplantation, whereas all mice in the BMT group survived during the study period (**Figure 1B**). In the GvHD group, all mice developed aGvHD symptoms since day 14, including weight loss, diarrhea, hunching, and decreased activity. All mice died within 22 days after allo-HSCT. Log-rank test revealed that the differences of survival between BMT vs GvHD, BMT vs PBS, and GvHD vs PBS groups were all statistically significant (*P*<0.05, **Figure 1B**). From day 3 after transplantation, the body weight of all mice in each group began to decrease as shown in Figure 1C. On day 21, the body weight of GvHD group was significantly lower than that of BMT group (N = 4 GvHD mice and N = 20 BMT mice, *P*<0.0001) (**Figure 1C**). Complete donor chimerism was achieved in both GvHD mice (**Figure 1D**) and BMT control mice (data not shown). Kinetics of WBC, Hgb, and platelet counts after HSCT were shown in **Figure 1E–G**. In the GvHD group, hematopoiesis in all three lineages was suppressed due to aGvHD from day 14. The differences between two treatment groups were statistically significant (*P*<0.01 for WBC, Hgb, and platelet, n = 4). 14 and 21 days after transplantation, the count of mononuclear cells (MNCs) in each tibia was examined. On day 14, there were significantly fewer MNCs in the GvHD group than that in the BMT group (1.10±0.21×10^7^ vs 1.71±0.032×10^7^, *P* = 0.0012, n = 4, **Figure 1H**). On day 21, hematopoiesis (MNCs) was further suppressed in the GvHD group compared with BMT group (0.58±0.14×10^7^ vs 2.14±0.38×10^7^, *P* = 0.0003, n = 4, **Figure 1H**).

We also examined the effects of aGvHD on absolute count of Lin^−^CD48^−^CD150^+^ cells in tibia and the percentage of Lin^−^CD48^−^CD150^+^ cells in MNCs. The results showed that on 14 day, either absolute number of Lin^−^CD48^−^/CD150^+^ cells (6.61±0.754×10^4^ vs 12.742±0.258×10^4^, *P*<0.0001, n = 4), or percentage of Lin^−^CD48^−^CD150^+^ cells in MNCs (0.6175±0.0338% vs 0.745±0.0648%, *P* = 0.013, n = 4) was significantly lower in the GvHD group vs BMT group. On day 21, while Lin^−^CD48^−^CD150^+^ cells increased in the BMT group, both absolute number of Lin^−^CD48^−^CD150^+^ cells and percentage of Lin^−^CD48^−^CD150^+^ cells in MNCs decreased in the GvHD group. The differences between GvHD and BMT were statistically different (*P*<0.0001, both for absolute count and percentage of Lin^−^CD48^−^CD150^+^ cells in MNCs, n = 4, **Figure 1I&J**). Another set of experiment for figure 1 was shown as figure S2.

Histological analysis showed that inflammation and tissue injury were presented in multiple organs on day 14 in the GvHD mice, including liver, skin, and intestine with different severity. By contract, there was little evidence showing lymphocyte infiltration and tissue injury in the mice of BMT group (**figure S1**). Collectively, these data demonstrated that aGvHD was successfully induced in this MHC-haploidentical matched murine HSCT model.

### Hematopoietic niche is the major target of aGvHD in MHC-haploidentical murine HSCT model

**Figure 2:**
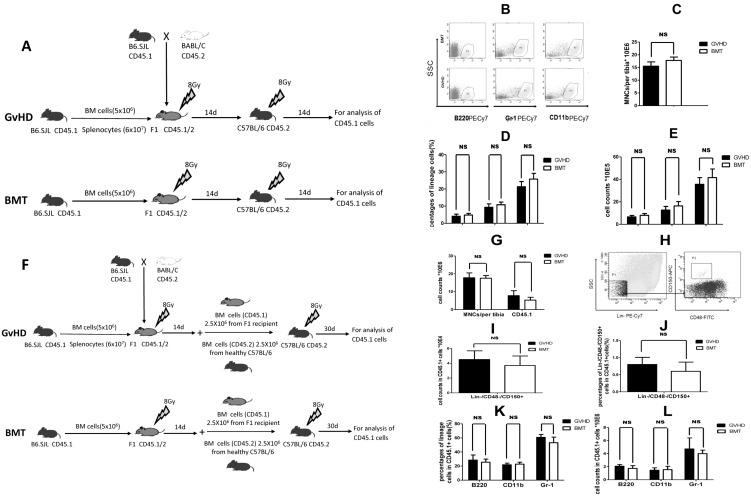
Hematopoietic cells derived from GvHD mice are competent for hematopoietic reconstitution (A–E) Continuous transplantation. Analyses were performed on day 14 after second transplantation. (A) Continuous HSCT: To evaluate the effects of GvHD on hematopoietic cell competency, lethally irradiated C57BL/6 (CD45.2) mice received BM cells(5×10^6^) from either BMT or GvHD recipient mice of [B6.SJL (CD45.1)→CB6F1 (CD45.1/2)] model at 14 days after first transplantation. (B) Representative flow cytometry analysis of.B cells (B220^+^), granulocytes (Gr-1^+^), and monocytes (CD11b^+^) in the recipient BM cells after continuous transplantation. (C) MNC count per tibia. (D) Percentages of B cells, granulocytes and monocytes in MNCs. (E) Counts of B cells, granulocytes and monocytes. (F–L) Competitive transplantation. Analyses were performed on day 30 after second transplantation. (F) Competitive transplantation to further evaluate the competency of hematopoietic cells in GvHD mice: 14 days after the first transplantation, BM cells (2.5×10^6^) from the transplanted mice [B6.SJL (CD45.1)→CB6F1 (CD45.1/2)] were mixed with equal amount (2.5×10^6^) of BM cells from healthy C57BL/6 mice (CD45.2), and transplanted into C57BL/6 recipients (CD45.2) after 8Gy radiotherapy. (G) Total MNC counts and CD45.1 positive cell counts per tibia. (H) Representative flow cytometry profile of HSCs (Lin^−^CD48^−^CD150^+^) analysis. (I) Absolute number of Lin^−^CD48^−^CD150^+^ cells in CD45.1 positive cell. (J) The percentage of Lin^−^CD48^−^CD150^+^ cells in CD45.1 positive cell. (K) and (L) Percentages and absolute number of B cells (B220^+^), granulocytes (Gr-1^+^), and monocytes (CD11b^+^), respectively. All tests were performed on day 30 after transplantation. Data are shown as mean ± SD and from 1 of 3 experiments with similar results. NS: no significant (n = 4, *t*-test).

It was reported that, in a MHC-mismatched murine GvHD model, hematopoietic niche, but not HSCs, was directly affected by aGvHD.[20] In order to test if this is also the case in our MHC-haploidentical murine GvHD model, we first tested if hematopoietic cells derived from the BM of GvHD mice were still competent in hematopoiesis by using continuous transplantation. To do so, lethally irradiated C57BL/6 mice (CD45.2) received BM from either BMT or GvHD mice in the [B6.SJL (CD45.1) →CB6F1 (CD45.1/2)] model (**Figure 2A**). The results showed there were no significant differences in the MNC count per tibia (Figure 2B & C n = 4,*P* = 0.0849), the percentages (Figure 2D) of B cells (B220^+^), monocytes (CD11b^+^) and granulocytes (Gr-1^+^) in MNCs (n = 4,B220, *P* = 0.3878; CD11b, *P* = 0.2993; Gr-1, *P* = 0.0933), and the absolute counts (n = 4,B220, *P* = 0.2055; CD11b, *P* = 0.1957; Gr-1, *P* = 0.2615;)(Figure 2E) between recipients of BMT- and GvHD-affected BM on day 14 after second transplantation. Another set of experiment for figure 2B–E was shown as figure S3.

To further verify the competency of GvHD-affected hematopoietic cells, we performed competitive transplantation in syngeneic transplantation model. 14 days after the first transplantation, the transplanted mice (donor: B6.SJL, H-2^b^ phenotype CD45.1; recipient: CB6F1 H-2^b/d^, phenotype CD45.1/2) were sacrificed. MNCs from transplanted mice were mixed with equal amount of MNCs from healthy C57BL/6 mice (H-2^b^, phenotype CD45.2). Total number of 5×10^6^ cells per mouse was implanted into C57BL/6 recipients ((H-2^b^, phenotype CD45.2) after 8Gy TBI (**Figure 2F**). The results demonstrated that, on day 30 after transplantation, there were no difference in the MNC count (n = 4, *P* = 0.08544) and CD45.1^+^ absolute value/per-tibia (n = 4, *P* = 0.16747) (Figure 2G), Lin^−^/CD48^−^/CD150^+^ cells absolute value (**Figure 2H and 2I**) (n = 4, *P* = 0.3918), percentage of Lin^−^/CD48^−^/CD150^+^ cells in MNCs (**Figure 2J**) (n = 4, *P* = 0.2866), percentages (**Figure 2K**) (n = 4,B220, *P* = 0.5103; CD11b, *P* = 0.6001; Gr-1, *P* = 0.1397) and absolute number (**Figure 2L**) (n = 4,B220, *P* = 0.2135; CD11b, *P* = 0.7855; Gr-1, *P* = 0.4473)of B cells (B220^+^), monocytes (CD11b^+^) and granulocytes (Gr-1^+^), between recipients of BMT- and GvHD-affected BM. These results indicate that hematopoietic cells from GvHD-affected BM retained functional competency to reconstitute hematopoiesis in a healthy hematopoietic niche. But the effects of GvHD on long-term HSCs could not be fully excluded due to the short follow-up post continuous transplantations. To further illuminate the effects of GvHD on HSCs, sufficient follow-up post continuous transplantation or sorted HSCs for continuous transplantation will be done.

**Figure 3:**
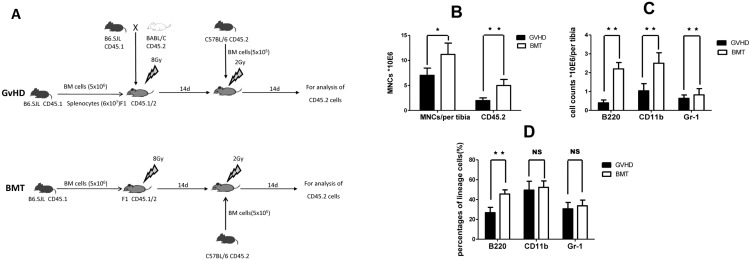
Impairment of BM hematopoietic niche during GvHD. (A) Re-transplantation: In order to evaluate the effects of GvHD on BM niche, recipient mice in the GvHD and BMT groups received a second transplantation from healthy C57BL/6 BM cells (CD45.2, 5×10^5^) after 200cGy TBI on days 14 after first transplantation. Hematopoiesis was analyzed on day 14 after re-transplantation. (B) Count of MNCs per tibia (left bars) and the percentage of C57BL/6 donor-derived CD45.2^+^ cells in MNCs (right bars). (C) Absolute number of donor-derived B cells (CD45.2^+^/B220^+^), monocytes (CD45.2^+^/CD11b^+^) and granulocytes (CD45.2^+^/Gr-1^+^). (D) Percentages of donor-derived B cells, monocytes and granulocytes in MNCs. Data are shown as mean ± SD and from 1 of 3 experiments with similar results. *P<0.05; **P<0.01; NS: no significant (n = 4, *t*-test).

In order to evaluate the effect of GvHD on hematopoietic niche, BM from healthy C57BL/6 donor mice (CD45.2) was retransplanted into either BMT or GvHD recipient mice of the [B6.SJL (CD45.1) →CB6F1 (CD45.1/2)] model on day 14, after 2 Gy TBI on day 13 (**Figure 3A**). We found that number of MNCs derived from retransplanted mice was decreased in GvHD recipient mice on day 14 after retransplantation (n = 4, *P* = 0.0202) (**Figure 3B**). The number of C57BL/6 donor-derived CD45.2^+^ cells was also significantly lower in the GvHD recipient group vs BMT group (n = 4, *P* = 0.0041) (**Figure 3B**). By examining all the lineages of hematopoiesis, we found that absolute number of B cells (CD45.2^+^/B220^+^, n = 4, *P* = 0.0001) and monocytes (CD45.2^+^/CD11b^+^, n = 4, *P* = 0.0052) (**Figure 3C**), as well as percentage of donor-derived B cells (n = 4, *P* = 0.0014) (**Figure 3D**), were lower in the GvHD recipient group than in the BMT group. Percentages of donor-derived granulocytes (CD45.2^+^/Gr-1^+^)and monocytes (CD45.2^+^/CD11b^+^), and absolute number of granulocytes, were not different between the GvHD recipient group and BMT group (**Figure 3C&D**). These results indicate that hematopoietic niche was impaired by GvHD.

### Destruction of vascular niche in aGvHD

**Figure 4:**
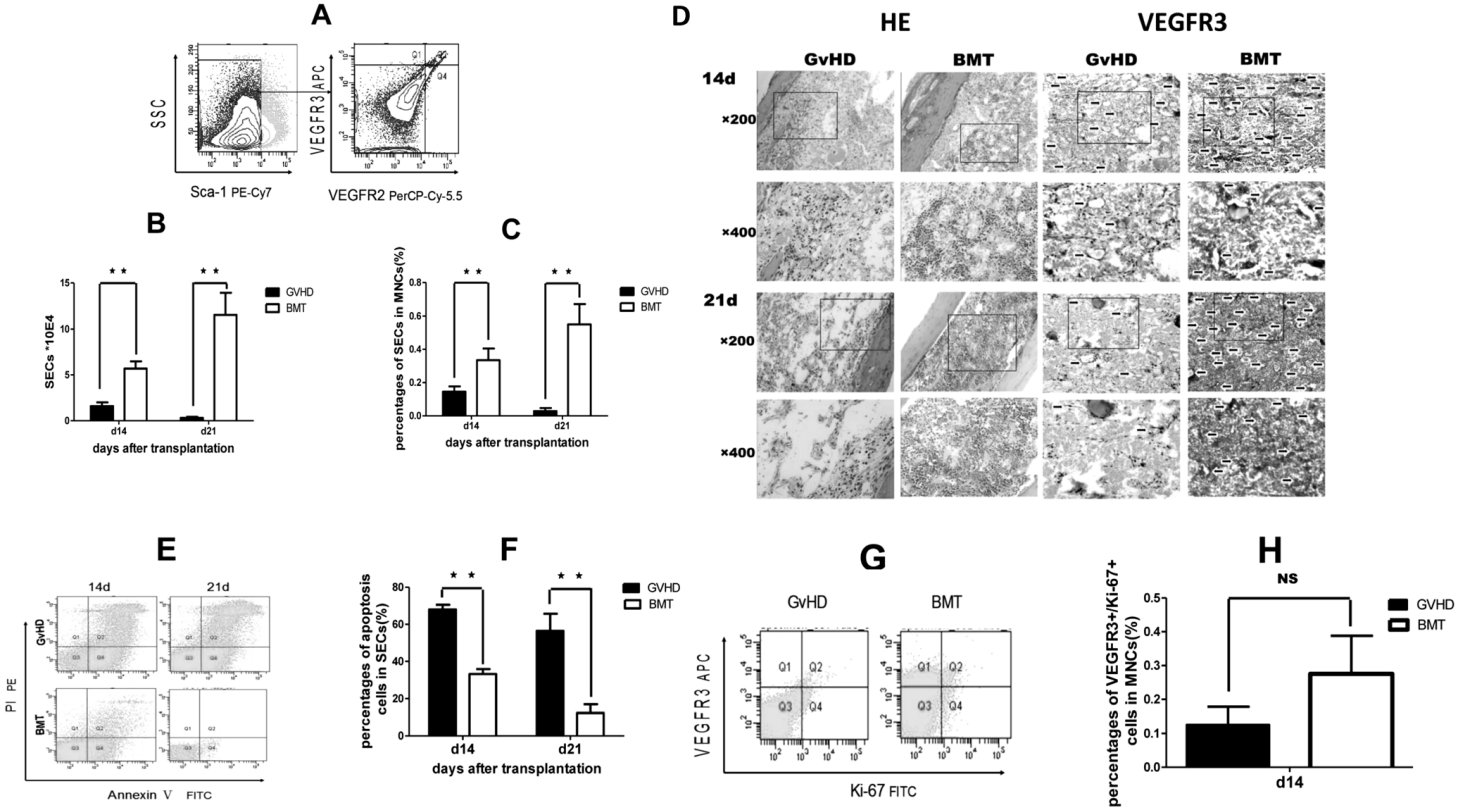
Destruction of vascular niche in acute GvHD. In order to verify vascular niche is the target of aGvHD, BM SECs from recipient mice were tested by flow cytometric and histological analysis. (A) Representative flow cytometry profile of SECs (Sca-1-VEGFR2+VEGFR3+). (B) Absolute number of BM SECs per tibia. (C) Percentages of BM SECs in MNCs. (D) HE and Immunohistochemistry analysis of disrupted vascular niche in acute GvHD vs BMT control. Arrows indicate SECS. (E) Representative flow cytometry analysis to assess apoptosis SECs. (F) Apoptosis of SECs, expressed by Annexin V^+^/PI^−^ and Annexin V^+^/PI^+^ to SSC^low^/VEGFR3^+^. (G and H) Proliferation assay of SECs by measuring Ki67 by flow cytometry and percentage of proliferating SECs (VEGFR3^+^/Ki-67^+^ cells) in MNCs. Data are shown as mean ± SD and from 1 of 3 experiments with similar results. **P<0.01 (n = 4, *t*-test).

To verify that vascular niche is the target of GvHD, BM SECs, from recipient mice with or without GvHD in the [B6.SJL (CD45.1) → CB6F1 (CD45.1/2)] model, were evaluated by flow cytometric and histological analysis. In our study, BM vascular SECs were characterized as VEGFR2^+^/VEGFR3^+^/Sca-1^−^ phenotype. 95% of VEGFR2^+^/VEGFR3^+^/Sca-1^−^ sorted SECs (**Figure 4A**) were VE-cadherin positive (data not shown). The results showed that, 14 days after transplantation, the absolute number of SECs per tibia in GvHD mice was significantly lower than that in the BMT group (1.5950±0.41×10^4^ *vs* 5.6950±0.78×10^4^, *P*<0.0001, n = 4) (**Figure 4B**). In GvHD mice, this number continued to decrease, and to 0.3234×10^4^ on day 21 after transplantation, while in the BMT group, the number returned to 11.55×10^4^ (*P*<0.0001). Similar results were found in the percentage of SECs in MNCs at 14 and 21 days after transplantation. The percentage of SECs in MNCs was significantly lower in the GvHD group than in the BMT group (*P* = 0.0026, *P*<0.0001), respectively (**Figure 4C**).

In order to confirm the SECs damage in GvHD mice, we performed histological analysis of the BM samples 7, 14, and 21 days after transplantation. The BM hyperplasia and changes of sinusoids and vascular endothelial cell were evaluated with HE and VEGFR3 staining of BM samples from the GvHD and BMT groups. BM hyperplasia was inhibited in the GvHD group 14 and 21 days after transplantation (Data not shown). At 14 days after transplantation, the number of nucleated cells and sinusoids was significantly reduced in GvHD mice compared with BMT mice, vacuolar and swollen sinuses with destroyed closed-construction could be found more often in GvHD vs BMT mice. At 21 days after transplantation, number of nucleated cells was further reduced, endothelial cells were seen occasionally, and sinusoid structure could hardly be recognized in GvHD mice. In the BMT group, however, more active hyperplasia was observed —marrow actively proliferated and flat-like sinusoids could be seen (**Figure 4D** **, HE**). As shown in figure 4D (**VEGFR3 staining**), the number of vascular endothelial cell was reduced obviously in GvHD group than in BMT group. At 21 days after transplantation, the vascular endothelial cells were further reduced than on 14 days after transplantation in GvHD group, while in the BMT group, the endothelial cells recovered in some extent at 21 days rather than 14 days after transplantation.

In order to determine how SECs were affected by GvHD, we examined the apoptosis and the proliferation capacity of SECs. The result demonstrated that, 14 and 21 days after transplantation, apoptosis of SECs, expressed by Annexin V^+^ (Annexin V^+^PI^−^and Annexin V^+^/PI^+^) to SSC^low^/VEGFR3^+^, was significantly higher in the GvHD group vs BMT group, 68.05±2.503% vs 33.250±2.67% at day 14 (n = 4, *P*<0.0001), and 56.450±9.237% vs 12.30±4.643% at day 21(n = 4, *P*<0.001) (**Figure 4E&F**), respectively. The proliferation capacity of SECs was evaluated by Ki-67 expression. 14 days after transplantation, the percentage of VEGFR3^+^/Ki-67^+^ cells in MNCs was no significant difference to the BMT group (0.125±0.053% vs 0.275±0.113%, *P* = 0.053, **Figure 4G&H**). Overall, these data demonstrated that vascular niche was destroyed during GvHD through the induction of apoptosis, not through affecting the proliferation capacity of SECs

### Dysfunction of vascular niche in aGvHD

**Figure 5:**
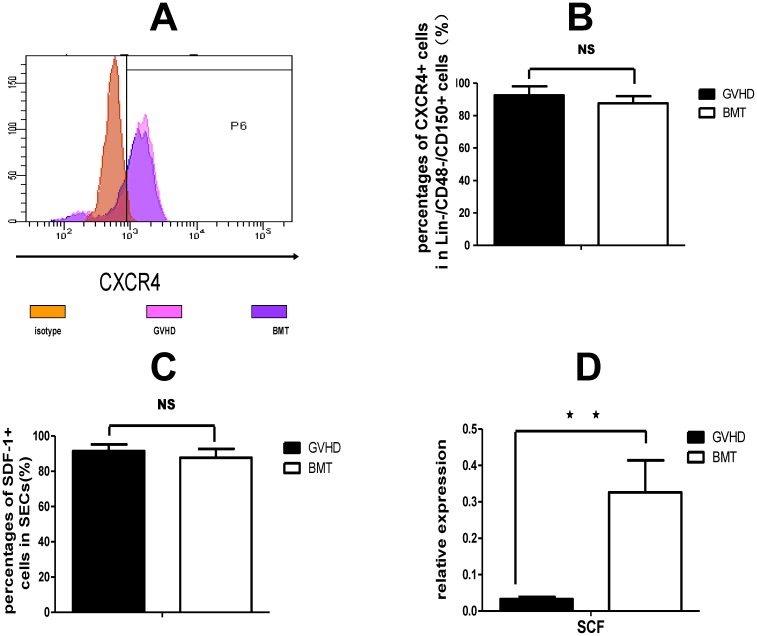
Dysfunction of vascular niche in acute GvHD. To evaluate the expression of CXCR4/SDF-1 and SCF/c-Kit in BM cells, flow cytometry and RT-PCR analysis were performed at 14 days after transplantation. (A) Representative flow cytometry profile of CXCR4 expression in Lin^−^CD48^−^CD150^+^ cells. (B–D): Analysis of CXCR4/SDF-1 and SCF/c-Kit pathways. (B) Percentage of CXCR4^+^ cells in HSCS. (C) Percentage of SDF-1^+^ cells in VEGFR3^+^ endothelial cells. (D) RT-PCR for measuring the expression of SCF in sorted BM SECs. Data are shown as mean ± SD. Experiments were performed at least twice. *P<0.05; **P<0.01; NS: no significant (n = 4 for CXCR4/SDF-1 analysis and n = 3 for SCF/C-Kit analysis, *t*-test).

In order to characterize the functional alterations of GvHD-impaired vascular niche, we performed flow cytometry and RT-PCR analysis of CXCR4/(stromal-derived factor 1) SDF-1 and stem cell factor (SCF)/c-kit in BM cells derived from GvHD vs BMT mice. SDF-1 expression in BM endothelial cells was analyzed on SSC^low^ VEGFR3^+^ cells and CXCR4 expression on HSCs was detected on LIN^−^CD48^−^CD150^+^ cells by flow cytomerty. SCF receptor (c-Kit) is expressed in HSCs, and SCF/c-Kit is one of the most important pathways that influence the renewal and differentiation of HSCs. [24] SCF expression was detected with RT-PCR analysis in sorted BM SECs. The SDF-1/CXCR4 pathway, however, regulate HSCs by mediating their homing and mobilization.[25] Similar to the data in **Figure 1J**, at day 14 after transplantation, percentage of Lin^−^/CD48^−^/CD150^+^ cells in BM MNCs was significantly lower in the GvHD group vs BMT group (0.6175±0.033% vs 0.745±0.0648%, n = 4, *P* = 0.013). However, there was no difference in the percentage of CXCR4^+^ cells in Lin^−^/CD48^−^/CD150^+^ cells between GvHD and BMT mice (n = 4, *P = *0.2159) (**Figure 5A&B**), nor the percentage of SDF-1^+^ cells in VEGFR3^+^ endothelial cells(n = 4, *P = *0.3504, **Figure 5C**). These results indicate that, in the GvHD mice, impaired hematopoietic niche may not affect HSCs and hematopoiesis via SDF-1/CXCR4 pathway.

We then investigated if SCF/c-kit pathway was affected in the GvHD mice. The results of RT-PCR showed that, on day 14 after transplantation, SCF expression in sorted BM SECs from the GvHD mice was much lower than that from the BMT mice (0.0329±0.138 vs 0.3261±0.006, n = 3, *P*<0.001). (**Figure 5D**). These results suggested that aGvHD may affect hematopoietic cell proliferation/differentiation and hematopoiesis by affecting the SCF/c-Kit pathway. Since SECs, the cells of vascular niche, are the major cells secreting SCF, it was indicated that vascular niche may be the target that mediates the hematopoietic impairment in aGvHD.

We then investigated the changes of VEGF in serum and BM microenvironment, since VEGF plays an important role in promoting the growth of SECs, and can be secreted by SECs. Our results showed that concentrations of VEGF in BM flushing (**Table 1** **, Table S2**) on 14 days after transplantation were significantly lower in the GvHD mice than in the BMT control mice (*P* = 0.0071, n = 3). VEGF concentration was also significantly lower in the serum from the GvHD mice 7 and 14 days after transplantation (*P* = 0.0021 and *P*<0.0001, respectively; n = 3) (**Table 1** **, Table S2**), which was consistent with the finding of reduced number of SECs in the GvHD BM. Taken together, these results further confirmed that the vascular niche was impaired in the aGvHD model.

**Table 1:** Concentration of VEGF in BM and serum.

	BM (ng/ml)		Serum (ng/ml)	
	GVHD	BMT	GVHD	BMT
+7 day	32.84±8.76	51.08±13.18	125.18±4.6	153.52±5.17
*P* value	0.1166		0.0021	
+ 14 day	55.64±9.23	125.70±22.09	149.16±4.71	229.92±3.84
*P* value	0.0071		< 0.0001	

Data are shown as mean ± SD and from 1 of 3 experiments with similar results. (n = 3, *t*-test).

### Donor CD4^+^ T cells mediate vascular niche damage in aGvHD

**Figure 6:**
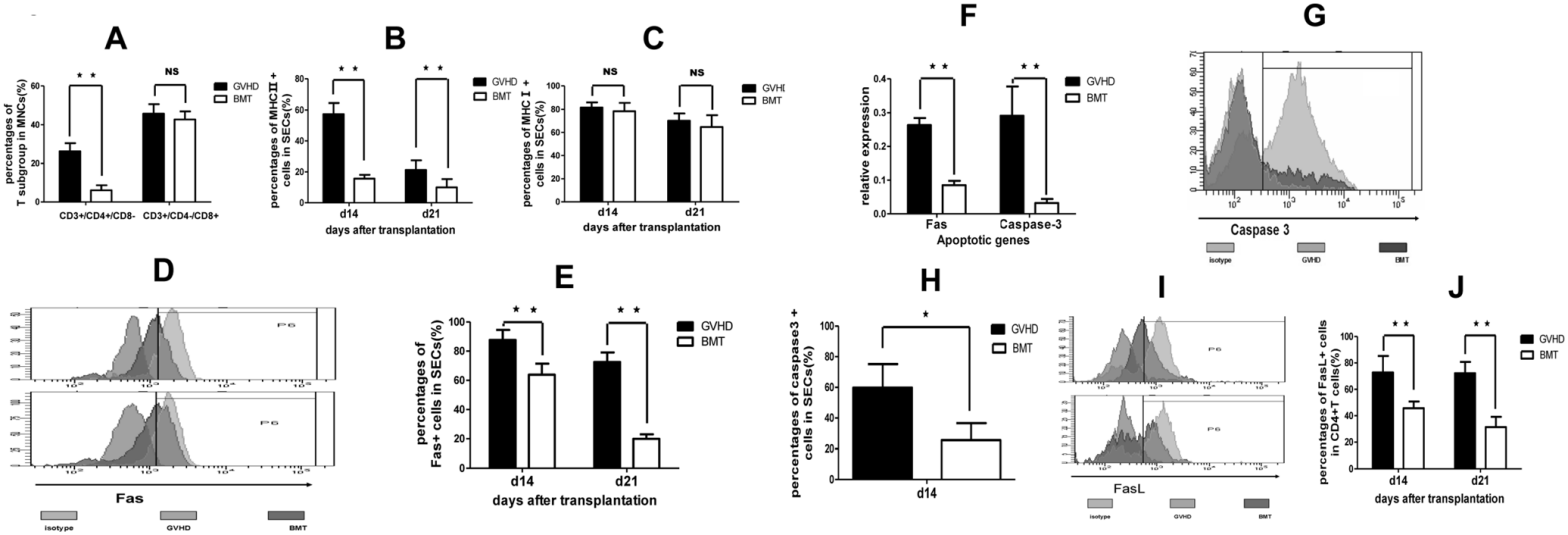
CD4^+^ T-cell mediated vascular niche damage in GvHD. (A) Percentages of CD4^+^ and CD8^+^ donor T cells in MNCs in BM at 14 days after transplantation. (B) MHC-II expression in BM SECs. (C) MHC-I expression in BM SECs. (D) Representative flow cytometry profile of Fas expression in BM SECs. (E) Percentages of Fas^+^ cells in SECs. (F) Expression of Fas and caspase-3 in BM SECs measured by RT-PCR. (G) Representative flow cytometry profile of caspase-3 expression in BM SECs. (H) Percentages of caspase-3 positive cells in SECs. (I) Representative flow cytometry of FasL in donor CD4^+^ T cells. (J) Percentages of FasL^+^ CD4^+^ donor T cells in MNCs. Data are shown as mean ± SD and from 1 of 3 experiments with similar results (Only one experiment for caspase-3 expression). **P<0.01; NS: no significant (n = 4, *t*-test).

Because donor T cells are the direct effector cells of aGvHD, we hypothesized that the destruction of vascular niche in recipient mouse may be mediated by donor T cells. To confirm this, we first examined the CD4^+^ and CD8^+^ donor T cells in BM. The results showed that, 14 days after transplantation, CD4^+^ T cell percentage was higher in the GvHD group than in the BMT group (25.94%±5.01 vs 6.83±1.99%, n = 4, *P* = 0.0004). No difference was noted in CD8^+^ T cell percentages between two groups (45.775%±4.96 vs 42.75%±4.21, n = 4 *P* = 0.3883) (**Figure 6A**).

Since CD4^+^ and CD8^+^ cells exert their cytotoxic activity through the ligation of MHC class II and I on target cells, respectively, we next assessed the MHC class I and II expression on BM cells from mice in the GvHD vs BMT groups. The results demonstrated that, 14 day after transplantation, MHC-II expression in BM SECs was significantly higher in the GvHD group than in the BMT group (n = 4, *P*<0.0001, **Figure 6B**). At the same time points, there was no difference in MHC-I molecule expression in BM SECs between the GvHD and BMT groups (*P* = 0.4707, **Figure 6C**). Similar results were found 21 days after transplantation (**Figure 6B&C**). These results suggested that the vascular niche destruction in GvHD mice was mediated by the cytotoxicity of donor CD4^+^ T cells rather than CD8^+^ T cells.

Recent studies have shown a role for the Fas-FasL pathway in GvHD mortality. In our study, 14 and 21 days after transplantation, Fas^+^ expression on BM SECs was significantly higher in the GvHD than in the BMT group (87.6±6.98% vs 63.9±7.53% at day 14, *P* = 0.0036; 72.5±6.65% vs 26.6±3.09% at day 21, *P*<0.0001; n = 4, **Figure 6D&E**). The Fas overexpression on SECs in the GvHD group was further confirmed by RT-PCR: 14 days after transplantation, relative expression of FAS gene in GvHD group was 0.2536±0.021 vs 0.0852±0.013 in the BMT group (n = 3, *P*<0.001, **Figure 6F**). In addition, caspase-3 expression was also higher in the GvHD group than in the BMT group, 0.2910±0.086 vs 0.0323±0.013 (n = 3, *P*<0.001, **Figure 6F**). The expression of caspase-3 was further confirmed with flow cytometry. Caspase-3 expression on BM SECs was significantly higher in the GvHD than in BMT group (59.75±15.42% vs 25.55±11.05%, *P* = 0.011285; n = 4, **Figure 6G&H**).

Because donor T cells are the effectors that mediate GvHD, we monitored FasL expression in both CD4^+^ and CD8^+^ T cells. We found that in the GvHD group, 14, 21 days after transplantation, CD4^+^ T cells had higher FasL expression than that in the BMT group (72.8±6.24% vs 45.72±2.53%, at day 14, *P* = 0.0002, and 72.2±8.61% vs 31.35±7.84% at day 21, *P* = 0.0004, n = 4, respectively, **Figure 6I&J**). Slight higher expression of FasL was found in CD8^+^ T cells 14 and 21 days after transplantation. Together, these results suggested that SECs were destructed by donor CD4^+^ T cells in the aGvHD mice. The SECs apoptosis was induced through Fas/FasL pathway.

## Discussion

Suppression of hematopoiesis during aGvHD has long been observed in both clinical and experimental studies. [23] In an MHC-mismatched murine GvHD model, Shono, et al reported that the disrupted hematopoiesis was not caused by direct elimination of HSCs in GvHD, but instead, was due to GvHD-impaired osteoblastic cells in the BM niche, which consequently failed to support the reconstitution of hematopoiesis, mainly B lymphopoiesis. [20]

There are two major hematopoietic niches in BM microenvironment to support hematopoiesis, one is endosteal niche, and the other is vascular niche. It was reported that osteoblast cells were the target of aGvHD; however, it's not clear whether vascular niche was also the target of aGvHD. In our study, we identified SECs, the cells of vascular niche, as a novel target for aGvHD in an MHC-haploidentical murine aGvHD model, which was destructed by donor CD4^+^ T cells through Fas/FasL pathway. Our data also indicated that hematopoietic cells were not directly affected by aGvHD, as evidenced by data that hematopoietic cells derived from GvHD mice had normal ability to reconstitute hematopoiesis in recipient mice. In contrast, hematopoietic cells from healthy donor mice failed to reconstitute hematopoiesis in the aGvHD recipient mice. These results, together with the direct evidence that vascular niche was damaged in the aGvHD mice; suggest that impaired vascular niche failed to support hematopoietic reconstitution by donor hematopoietic cells.

Our data demonstrated that the donor CD4^+^, but not CD8^+^ T cells, were responsible for the cytotoxicity against SECs, through Fas/FasL apoptotic pathway. In line with this, MHC class II expression was upregulated in SECs, suggesting that the destruction of SECs by CD4^+^ T cells was via MHC class II-TCR interactions. This differs from the results shown in the MHC-mismatched murine HSCT model, where the donor CD4^+^ T-cells exerted the destruction of osteoblasts independent of class II–T-cell receptor interaction. The differences may be due to different HSCT models used in the studies.

Cytotoxic T lymphocytes (CTL), including CD4^+^ and CD8^+^ CTLs, are the main effector cells of aGvHD that mediate cytotoxic function against host cells via Fas/FasL and perforation/granzyme pathways.[26] CD4^+^ CTLs exert their cytotoxicity mainly through the Fas/FasL pathway, whereas CD8^+^ CTL mainly relies on perforin/granzyme pathway to cause damage to the target cells. The Fas/FasL activation ultimately leads to caspase-3 activation, the final executor in caspase-dependent apoptosis pathway. High Fas and caspase-3 expression in BM SECs in the GvHD mice, and high FasL expression in the donor CD4^+^ T cells, suggested that CD4^+^ T cells induce endothelial cell apoptosis via the Fas/FasL pathway.

As a major component of BM vascular niche, SECs can be identified via co-expression of VEGFR2 and VEGFR3 and the absence of Sca-1.[10] It was reported that inhibition of BM vasculogenesis by an anti-VE-cadherin antibody resulted in suppressed multilineage hematologic recovery following myelosuppression, confirming that BM EC-mediated VEGFR2 signaling is essential for normal hematopoietic regeneration following myelosuppression *in vivo*.[17] Soluble factors produced by BM SECs, including SCF and PTN, are also responsible for HSC self-renewal and regeneration *in vivo*.

In this study, the results suggested that SCF/c-kit axis, but not SDF-1/CXCR4 axis, was inhibited during aGvHD. These results further provided supporting evidence that vascular niche is impaired in aGvHD. As reported by Rafii *et al*, [13] human BM endothelial cells (ECs) produce several hematopoietic cytokines, including SCF, to support proliferation and differentiation of human CD34^+^ cells in culture. According to the model proposed by Heissig *et al*, [27] soluble SCF released by ECs facilitates translocalization of HSCs to the BM vascular niche for proliferation and differentiation, while re-establishment of BM vasculature is the key process in thrombopoiesis recovery.[15] These studies suggested an important function for BM vascular niche in supporting hematopoiesis and hematopoietic regeneration *in vivo*.

Interestingly, in our study, VEGF level in serum and local BM microenvironment was reduced in aGvHD mice. VEGF is a highly specific mitogen for SECs, and is known to promote the proliferation of endothelial cells. A number of studies have shown that the serum level of VEGF is negatively correlated to GVHD severity. [28], [29] It was also found that vascular endothelial cells can secrete VEGF to prevent cell apoptosis, and to protect cell basement membrane and cell plasticity. [30] In our study, we found that the serum VEGF concentration in serum and local BM microenvironment was lower in GvHD mice after MHC-haploidentical transplant, which is consistent with the reduced number of SECs in GvHD BM.

In conclusion, we have identified SECs as a novel target for aGvHD in an MHC-haploidentical murine HSCT model. Therefore, protection of SECs from apoptosis by using cytoprotective agents might be an effective approach to antagonize aGvHD-related hematopoietic failure. It was reported that rapamycin (sirolimus), an mTOR inhibitor, can inhibit cell apoptosis. [31], [32] and is currently used in the prophylaxis of aGvHD. It would be interesting to investigate whether this agent can specifically protect SECs from aGvHD-induced apoptosis, and in turn prevent hematopoietic failure in aGvHD. Systemic infusion of genetic engineered SECs that might provide donor T-cell-mediated cytotoxicity resistance and promote BM vascular niche recovery and hematopoiesis could be another interesting strategy.

## Supporting Information

Figure S1 Histological analysis of aGVHD targets. In GvHD mice, the liver had more inflammatory cell infiltration, and the hepatocytes were swollen and fuzzy. Severe inflammatory cell infiltration was seen in the skin. The small intestine was also infiltrated with inflammatory cells, with intestinal villi fractures.
(TIFF)

Figure S2 **Suppression of hematopoiesis during GvHD (another repeated experiment for figure1B–1J).** (A) Survival of mice receiving HSCT with donor bm plus splenocytes or donor BM only (*P*<0.05, Log-rank test). (B) Body weight of mice receiving HSCT (N = 20 in each group on day 3; n = 4 in GvHD and n = 20 in BMT respectively on day 21 post-transplantation). (C) Engraftment of donor-derived cells after HSCT in a GvHD mouse. (D–F) Kinetics of WBC, Hgb, and platelet counts after HSCT. (G) MNCs count on day 14 and day 21 after HSCT. (H) Count of Lin^-^/CD48^−^/CD150^+^ cells after HSCT. (I) Percentage of Lin^−^/CD48^−^/CD150^+^ cells in MNCs after HSCT. Data are shown as mean ± SD. *P<0.05; **P<0.01 (n = 4, *t*-test)
(PDF)

Figure S3 **Hematopoietic cells derived from GvHD mice are competent for hematopoietic reconstitution (Another repeated experiment for** **figure 2B–2E** **).** Continuous transplantation. Analyses were performed on day 14 after second transplantation. (A) Representative flow cytometry analysis of.B cells (B220^+^), granulocytes (Gr-1^+^), and monocytes (CD11b^+^) in the recipient BM cells after continuous transplantation. (B) MNC count per tibia. (C) Percentages of B cells, granulocytes and monocytes in MNCs. (D) percentages of b cells, granulocytes and monocytes in MNCs. Data are shown as mean ± SD. NS: no significant (n = 4, *t*-test).
(TIFF)

Table S1 Primers used for RT-PCR analysis.
(PDF)

Table S2 Concentration of VEGF in BM and serum (Another repeated experiment for Table 1).
(TIFF)
