# Supplementary figures and images for: Dysfunction of Bone Marrow Vascular Niche in Acute Graft-Versus-Host Disease after MHC-Haploidentical Bone Marrow Transplantation
Source: PLoS One. 2014 Aug 13;9(8):e104607. doi: 10.1371/journal.pone.0104607 (PMC4131885; doi:10.1371/journal.pone.0104607)

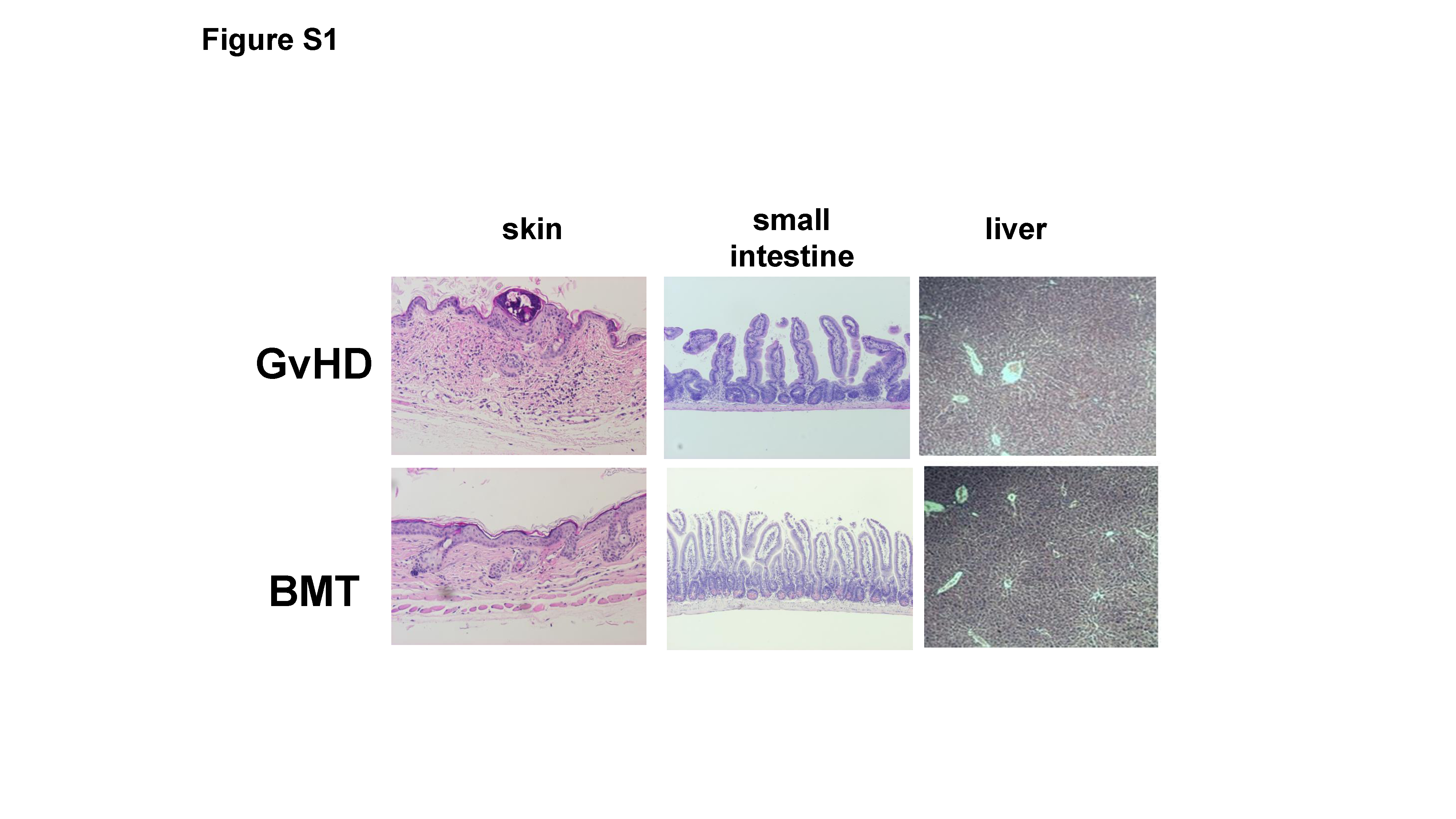

Supplement: Figure S1 — Histological analysis of aGVHD targets. In GvHD mice, the liver had more inflammatory cell infiltration, and the hepatocytes were swollen and fuzzy. Severe inflammatory cell infiltration was seen in the skin. The small intestine was also infiltrated with inflammatory cells, with intestinal villi fractures. (TIFF) [file pone.0104607.s001.tiff]

Figure S2

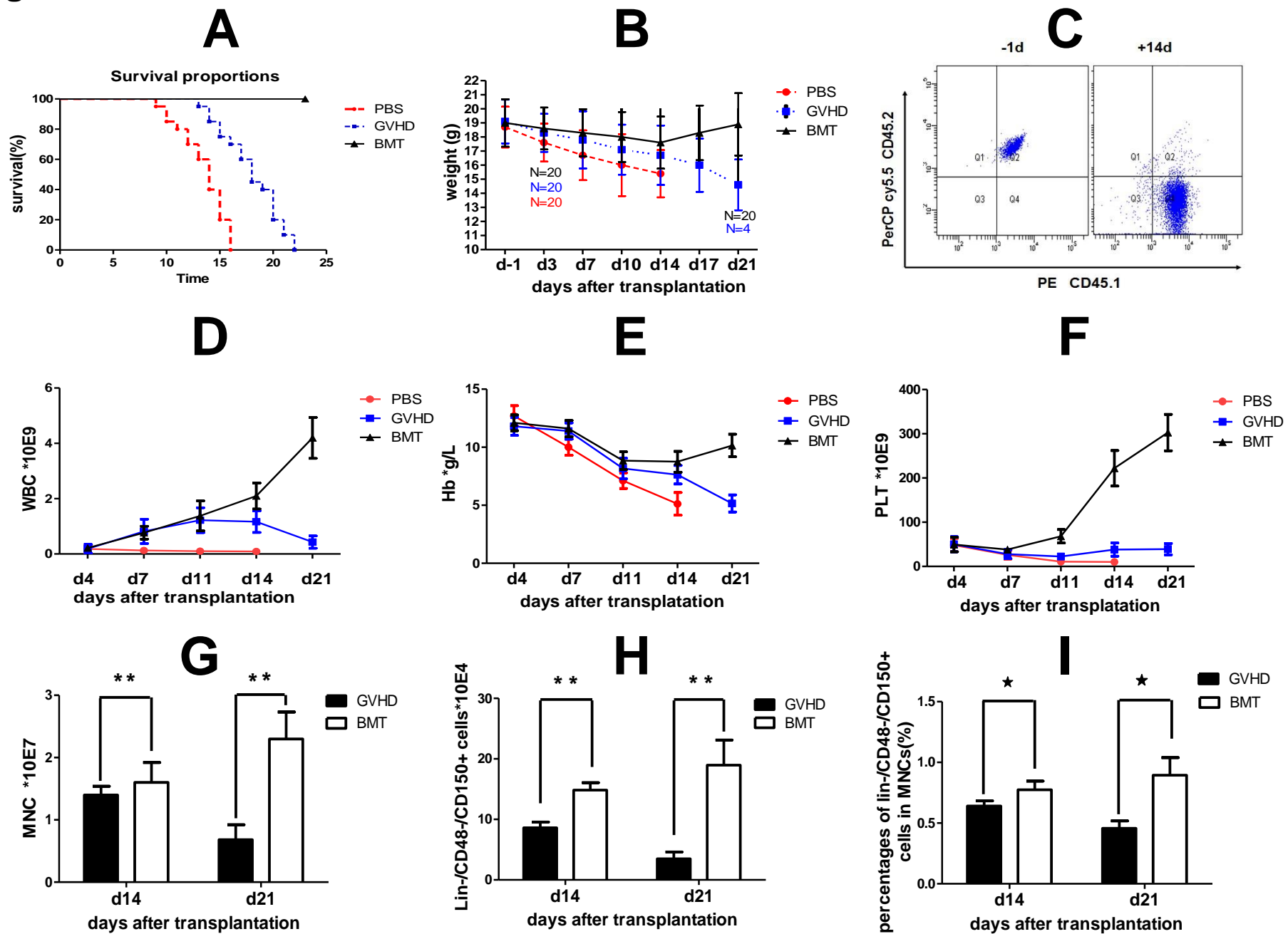

Supplement: Figure S2 — Suppression of hematopoiesis during GvHD (another repeated experiment for figure1B–1J). (A) Survival of mice receiving HSCT with donor bm plus splenocytes or donor BM only (P<0.05, Log-rank test). (B) Body weight of mice receiving HSCT (N = 20 in each group on day 3; n = 4 in GvHD and n = 20 in BMT respectively on day 21 post-transplantation). (C) Engraftment of donor-derived cells after HSCT in a GvHD mouse. (D–F) Kinetics of WBC, Hgb, and platelet counts after HSCT. (G) MNCs count on day 14 and day 21 after HSCT. (H) Count of Lin-/CD48−/CD150+ cells after HSCT. (I) Percentage of Lin−/CD48−/CD150+ cells in MNCs after HSCT. Data are shown as mean ± SD. *P<0.05; **P<0.01 (n = 4, t-test) (PDF) [file pone.0104607.s002.pdf]

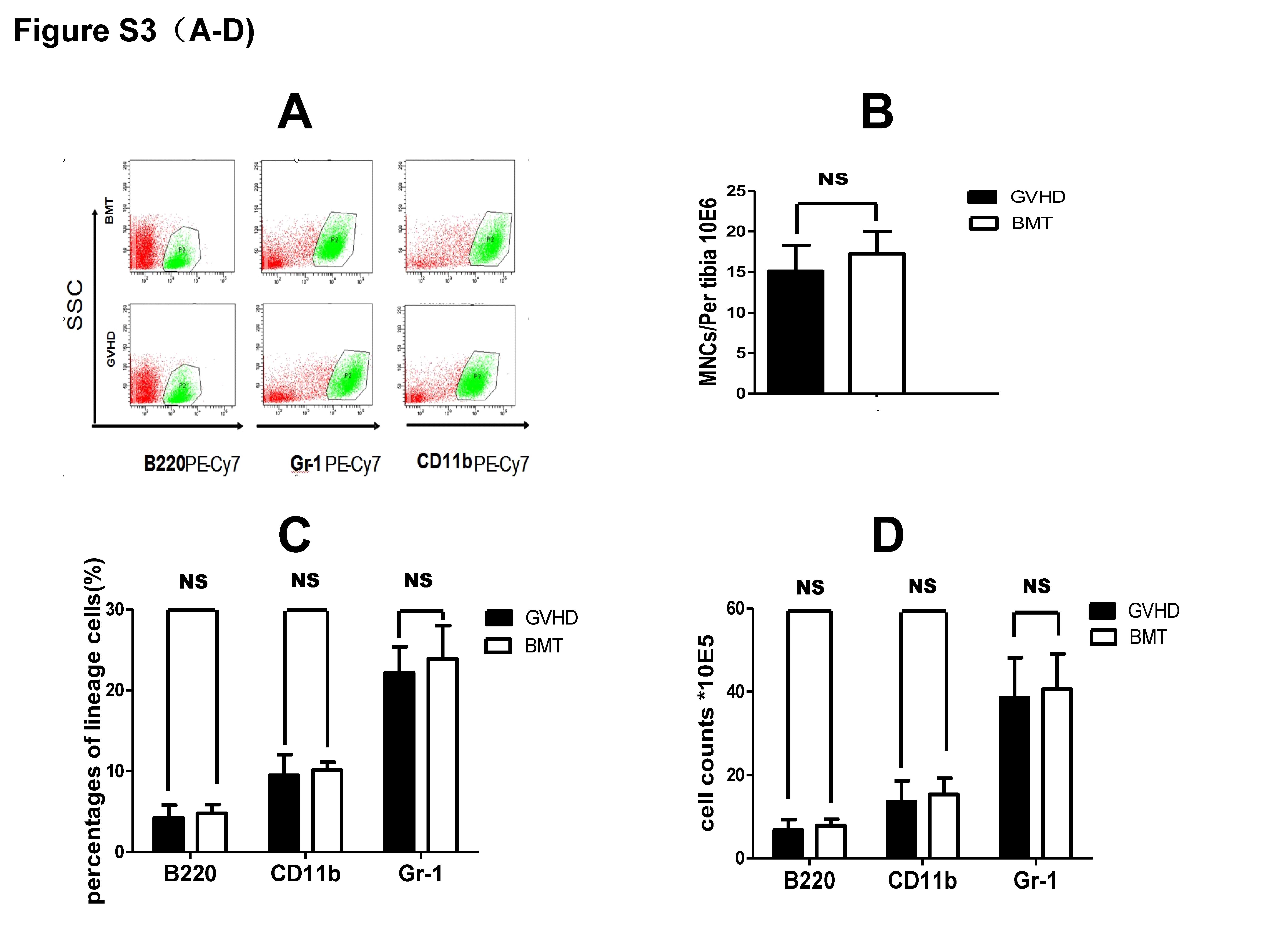

Supplement: Figure S3 — Hematopoietic cells derived from GvHD mice are competent for hematopoietic reconstitution (Another repeated experiment for figure 2B–2E ). Continuous transplantation. Analyses were performed on day 14 after second transplantation. (A) Representative flow cytometry analysis of.B cells (B220+), granulocytes (Gr-1+), and monocytes (CD11b+) in the recipient BM cells after continuous transplantation. (B) MNC count per tibia. (C) Percentages of B cells, granulocytes and monocytes in MNCs. (D) percentages of b cells, granulocytes and monocytes in MNCs. Data are shown as mean ± SD. NS: no significant (n = 4, t-test). (TIFF) [file pone.0104607.s003.tiff]
