# Supplementary material for: Dysfunction of Bone Marrow Vascular Niche in Acute Graft-Versus-Host Disease after MHC-Haploidentical Bone Marrow Transplantation
Source: PLoS One. 2014 Aug 13;9(8):e104607. doi: 10.1371/journal.pone.0104607 (PMC4131885; doi:10.1371/journal.pone.0104607)
Supplement: Table S1 — Primers used for RT-PCR analysis. (PDF) [file pone.0104607.s004.pdf]

Table S1. Primers used for RT-PCR analysis

	-Forward	-Rev
GAPDH	AAC TTT GGC ATT GTG GAA GG	TGT GAG GGA GAT GCT CAG TG
SCF	CGG GAA TCC TGT GAC TGA TAA	CTG TCA TTC CTA AGG GAG CTG
FasL	CAT CAC AAC CAC TCC CAC TG	TGA ATA CTG CCC CCA GGT AG
Caspase3	CTC ACA TAG CAG GGA GCA CA	GAA AAC CGT GAA GGC AAC AT
